# Two-omics data revealed commonalities and differences between Rpv12- and Rpv3-mediated resistance in grapevine

**DOI:** 10.1038/s41598-020-69051-6

**Published:** 2020-07-22

**Authors:** Giulia Chitarrini, Samantha Riccadonna, Luca Zulini, Antonella Vecchione, Marco Stefanini, Simone Larger, Massimo Pindo, Alessandro Cestaro, Pietro Franceschi, Gabriele Magris, Serena Foria, Michele Morgante, Gabriele Di Gaspero, Urska Vrhovsek

**Affiliations:** 1 Research and Innovation Centre, Fondazione Edmund Mach, via E. Mach 1, 38010 San Michele all’Adige, Italy; 2 Dipartimento di Scienze Agroalimentari, Ambientali e Animali, Università di Udine, via delle Scienze 208, 33100 Udine, Italy; 3 Istituto di Genomica Applicata, via Jacopo Linussio 51, 33100 Udine, Italy

**Keywords:** Genetics, Plant sciences

## Abstract

*Plasmopara viticola* is the causal agent of grapevine downy mildew (DM). DM resistant varieties deploy effector-triggered immunity (ETI) to inhibit pathogen growth, which is activated by major resistance loci, the most common of which are *Rpv3* and *Rpv12*. We previously showed that a quick metabolome response lies behind the ETI conferred by *Rpv3* TIR-NB-LRR genes. Here we used a grape variety operating *Rpv12*-mediated ETI, which is conferred by an independent locus containing CC-NB-LRR genes, to investigate the defence response using GC/MS, UPLC, UHPLC and RNA-Seq analyses. Eighty-eight metabolites showed significantly different concentration and 432 genes showed differential expression between inoculated resistant leaves and controls. Most metabolite changes in sugars, fatty acids and phenols were similar in timing and direction to those observed in *Rpv3*-mediated ETI but some of them were stronger or more persistent. Activators, elicitors and signal transducers for the formation of reactive oxygen species were early observed in samples undergoing *Rpv12*-mediated ETI and were paralleled and followed by the upregulation of genes belonging to ontology categories associated with salicylic acid signalling, signal transduction, WRKY transcription factors and synthesis of PR-1, PR-2, PR-5 pathogenesis-related proteins.

## Introduction

Downy mildew is one of the most destructive diseases of the grapevine, causing significant limitations on grape production in the absence of chemical protection of vineyards. Downy mildew is caused by the biotrophic oomycete *Plasmopara viticola* (Berk. And Curt) Berl. and Toni, which is native to North America and was introduced into Europe at the end of the nineteenth century. The European bunch grape (*Vitis vinifera* L.) does not normally activate the immune system in response to *P. viticola* with a few exceptions^1,2^. The introgression of resistant genes from other grape species (i.e. *V. rupestris*, *V. amurensis*, *V. cinerea*, *V. riparia* and *Muscadinia rotundifolia*) into the crop germplasm can alleviate the dependence of viticulture on the use of fungicides^3^.

The pathogen can cause serious damage to any green organ of the grapevine. *P. viticola* deploys a specialized structure called haustorium to establish a close interaction with grapevines and to leak nutrients from viable host cells^4,5^. *P. viticola* can feed on both susceptible and resistant grapevines and complete its life cycle on both hosts. Resistant grapevines are, however, able to detect the invading pathogen and operate defence responses. The initial phases of the infection are similar on both hosts, but mycelial growth is restricted in resistant hosts and sporangia are released at lower rates than in susceptible hosts. The similarity during early phases of infection suggests the presence of post-infection mechanisms of resistance, including callose deposition, cell wall-associated defence processes, accumulation of reactive oxygen species and hypersensitive response (HR) associated with necrosis, and production of phytoalexins and antimicrobial compounds^6–12^. These responses are paralleled by the up-regulation of genes coding for pathogenesis-related (PR) proteins^13^. The study of the defence response in resistant grape varieties can improve our understanding of the resistance mechanisms and shed light on the metabolic pathways involved. It is commonly believed that ETI requires a massive reallocation of energy and materials towards a multitude of actions against pathogen colonisation^14,15^, which reduce fitness in annuals^16,17^ and might be energetically costly in perennial crops grown under limiting conditions^18,19^.

We recently performed metabolomic analyses after pathogen inoculation in the grape variety ‘Bianca’ that carries a major resistance haplotype called *Rpv3-1*, leading to the identification of 53 metabolites associated with the defence response^20^. We decided to extend this investigation using another resistant grapevine that has a genetic background highly similar to ‘Bianca’. This grapevine shares a complete haploid complement with its parent ‘Bianca’ but not the resistance haplotype *Rpv3-1* at the *Rpv3* locus on chromosome 18 and it carries instead a different resistance gene—*Rpv12*—that is located on chromosome 14. We first identified a suitable genotype for this kind of experiment in an accession obtained in 2000 by P. Kozma and S. Hoffmann in Hungary from a cross between ‘Bianca’ and the breeding line SK77*-*4/5 and held in a germplasm repository in South Tyrol (Italy). The resistance haplotype at the *Rpv12* locus was initially introgressed from the Asian species *Vitis amurensis* and confers higher levels of downy mildew resistance than *Rpv3*-*1* when the resistance phenotype is quantitatively scored using the OIV452 descriptor^21^, but both genes trigger a similar ETI-dependent response^12,21^. The donor parent of the *Rpv12* resistance haplotype, SK77*-*4/5, was bred at the University of Novi Sad, Serbia^22^ by crossing *V. vinifera* ‘Traminer’ and the resistant variety ‘Kunbarat’, which descended from the interspecific hybridization of *V. amurensis* and *V. vinifera*. In our previous study in ‘Bianca’^20^, we focused on *Rpv3*-mediated metabolome changes upon pathogen inoculation with the aim to identify biomarkers of resistance. Recently, Eisenmann and coworkers^23^ performed a transcriptomic analysis in ‘Regent’, a resistant variety that operates the same *Rpv3*-mediated ETI response as does ‘Bianca’, showing that *Rpv3*-mediated changes in gene expression occur early after pathogen inoculation.

Technological developments in genomics, transcriptomics, proteomics and metabolomics have provided powerful tools for systems biology studies. Metabolomics is widely used for monitoring changes in response to the developmental program, to the environment and to biotic stresses^24^. In grapevine, metabolomics has been used to investigate several aspects of berry development and berry ripening^25–27^ or to study the effects of abiotic stresses^28–30^. More recent studies using grape leaves that had been inoculated with *P. viticola* have used metabolomic analyses to monitor specific classes of compounds or a limited number of molecules^31–33^. In our previous work^20^, we extended the number of compounds that are identified and quantified in grape leaves using a single analytical run. Transcriptomics refers to the global analysis of all transcribed genes in a certain organ. Transcriptomic analyses based on RNA-Sequencing (RNA-Seq) provide a comprehensive survey of gene expression changes occurring during the response to pathogen infections. Transcriptomic analyses have been performed on grape leaves undergoing defence responses to *P. viticola* in native American species^13^ and in *V. vinifera* introgression lines carrying *Rpv3-1*, *Rpv3-3*, or *Rpv10* resistance genes^23,34,35^. Using a single-omics approach it is possible to clarify only one aspect of cell metabolism. Multi-omics analyses offer a unifying view of the biological processes and have been applied to fruit crop species^36^ for inferring relationships between genes, transcripts, metabolites and phenotypes. Recent work has been done in grapevine by applying a multi-omics integrated approach to study drought stress, berry developmental changes, light exclusion, basal immunity and responses to *Erysiphe necator* infection^25,26,30,37,38^. Here we present two integrated omics data of *Rpv12*-mediated ETI responses to *P. viticola* in grapevine leaves and compare the results with our previous data^20^ and other literature reports^23,31^ in which the same two-omics were used separately for studying *Rpv3*-mediated responses.

## Results

### Impact of the defence response on leaf metabolome

**Figure 1:**
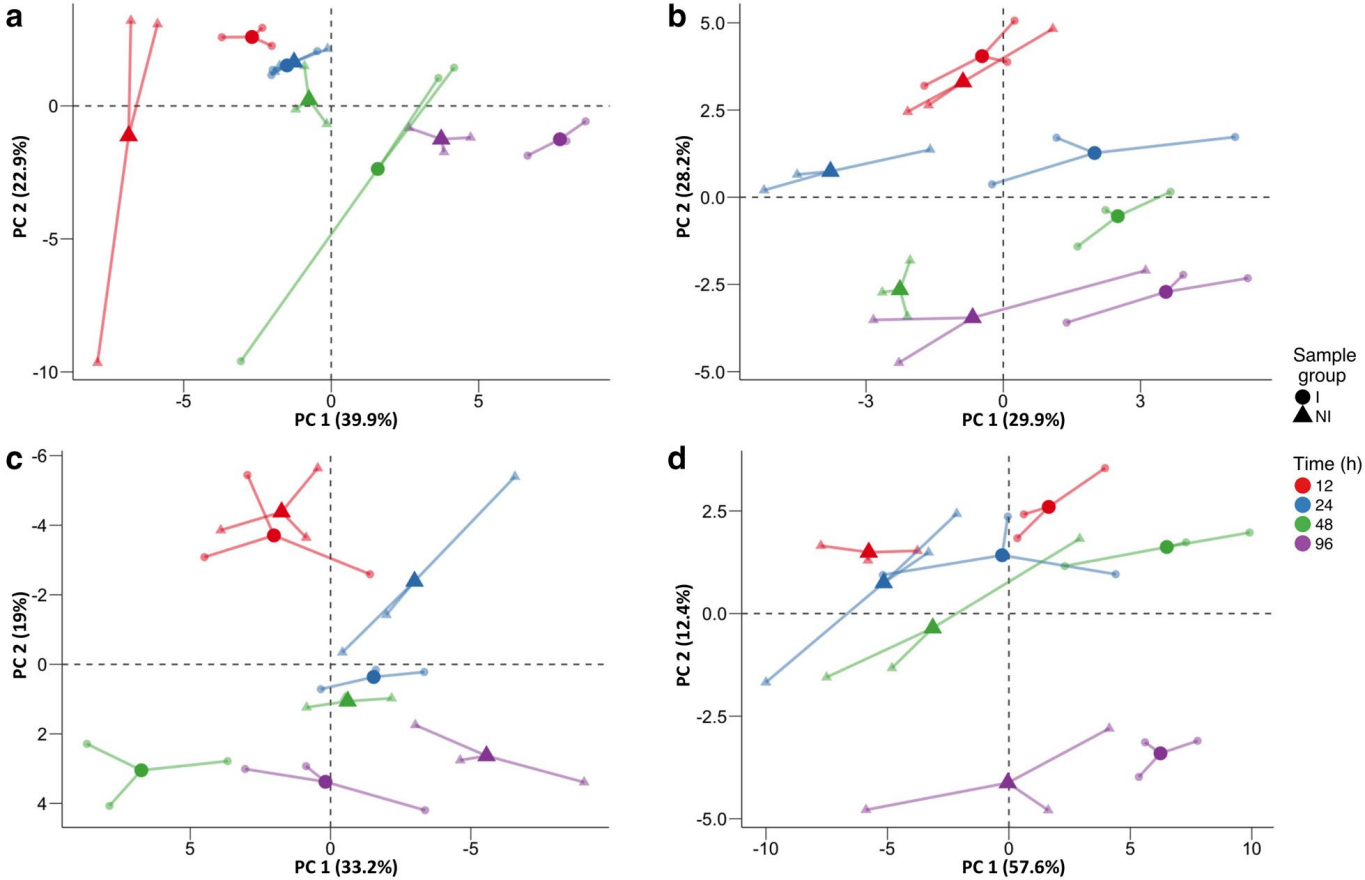
PCA of metabolite data in leaves undergoing *Rpv12*-mediated defence (inoculated black filled circle) and controls (black filled triangle). Three biological replicates are reported as small symbols (black filled circle, black filled triangle) and are linked to their mean values (large symbols, black filled circle, black filled triangle) by connectors (–). (**a**) Primary metabolites. (**b**) Lipids. (**c**) Phenols. (**d**) Volatile compounds. Different colours represent different time points: red filled circle, red filled triangle—12 hpi; blue filled circle, blue filled triangle—24 hpi; green filled circle, green filled triangle—48 hpi; violet filled circle, violet filled triangle—96 hpi.

We identified and quantified 175 metabolites in inoculated and control leaves. A complete list of these compounds and their concentration is reported in Supplementary Table S1, sorted into four classes of primary metabolites, lipids, phenols, and volatile compounds. Figure 1 illustrates a lower-dimensional picture of the distribution of samples undergoing *Rpv12*-mediated ETI responses and controls during the course of the experiment using a Principal Component Analysis (PCA). The first two components explained 62.8, 58.1, 52.2, and 70.0% of the observed variation for primary metabolites, lipids, phenols, and volatile compounds, respectively. The first component (PC1) revealed a separation between inoculated samples and controls at 12 and 96 h post infection (hpi) for primary metabolites and volatile compounds (Fig. 1a,d), at 24 and 48 hpi for lipids (Fig. 1b), and at 48 and 96 hpi for phenols (Fig. 1d). The second component (PC2) separated the samples according to the stage of incubation.

**Figure 2:**
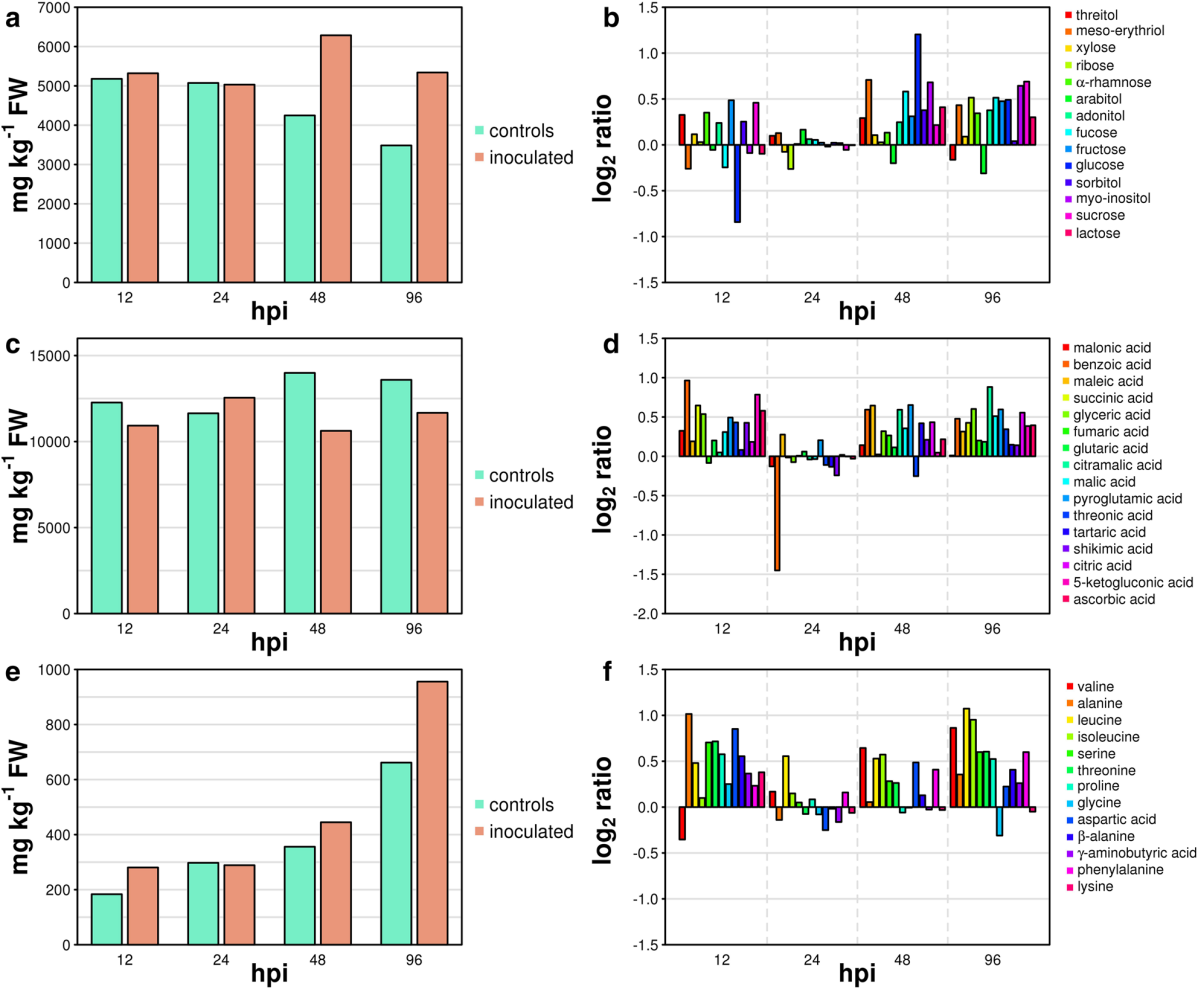
Major classes of primary metabolites in leaves undergoing *Rpv12*-mediated defence (inoculated) and controls. Cumulative concentration of (**a**) sugars, (**c**) organic and (**e**) amino acids. (**b**,**d**,**f**) Report the log_2_ ratio of the concentration of each compound in inoculated leaves versus controls.

**Figure 3:**
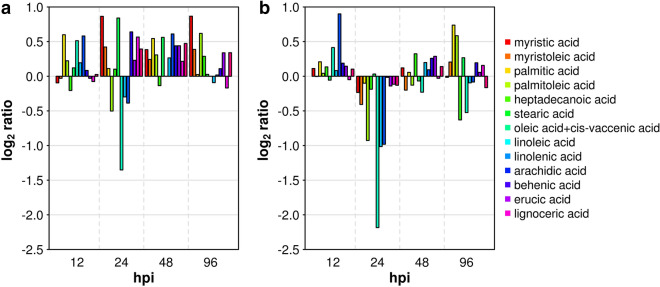
Differences in fatty acids between leaves undergoing (**a**) *Rpv12*- or (**b**) *Rpv3*-mediated defence. On the y-axis the  log_2_ ratio of the concentration of each compound in inoculated leaves versus controls is shown. Graph in (**b**) was plotted using raw data obtained from our previous paper^20^.

**Figure 4:**
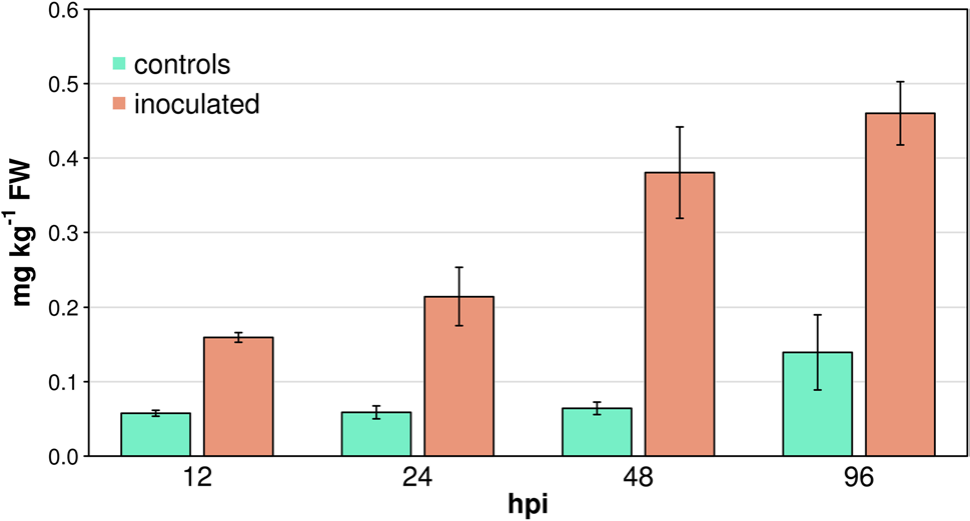
Concentration of benzaldehyde in leaves undergoing *Rpv12*-mediated defence (inoculated) and controls during the course of infection. Bars represent ± standard errors.

With regard to primary metabolism, we observed two distinct temporal patterns of activation. A rapid and transient increase in the concentration of some sugars and most of organic and amino acids was detected at 12 hpi (Fig. 2). A delayed and more sustained increase of almost all sugars, organic acids and amino acids occurred from 48 to 96 hpi (Fig. 2). We observed the least differences in primary metabolites between inoculated samples and controls at the stage of 24 hpi with the notable exception of benzoic acid that was greatly reduced in samples undergoing *Rpv12*-mediated defence (Fig. 2d). Benzoic acid is the precursor of salicylic acid (SA) in the phenylalanine ammonia-lyase (PAL) pathway^39^. We therefore assume that the benzoic acid pool is depleted at 24 hpi by a rapid conversion of this substrate to SA. The concentration of fatty acids also increased in samples undergoing *Rpv12*-mediated defence (Fig. 3a). Linolenic acid (18:3) was among the fatty acids with elevated concentration in samples undergoing *Rpv12*-mediated defence early at 12 hpi compared to controls (Fig. 3a) and this behaviour was in common with samples undergoing *Rpv3*-mediated defence (Fig. 3b) and with literature reports^40^. Linolenic acid is reported to activate NADPH–oxidase—an enzyme responsible for the production of reactive oxygen species (ROS)—and is important for the modulation of the strength of the HR^41^ if not responsible itself for the onset of the oxidative burst in programmed cell death (PCD)^42^. In a context of an elevated lipid metabolism (Fig. 3a), we observed a marked decrease in palmitoleic acic (16:1), linoleic acid (18:2), linolenic acid (18:3), and especially in oleic and vaccenic acids (18:1) at the stage of 24 hpi. It is noteworthy that the concentration of the same five fatty acids showed the most remarkable decreases in samples undergoing *Rpv3*-mediated defence at the same stage of incubation (Fig. 3b). Low levels of 18:1 fatty acids in Arabidopsis mutants, which are a consequence of an inactivating mutation in the SSI2 gene encoding an isoform of stearoyl-desaturase for the conversion of stearic acid (18:0) to oleic acid (18:1), cause constitutive SA-signalling and expression of PR proteins^43^. We also observed a general increase in the concentration of volatile compounds in samples undergoing *Rpv12*-mediated defence (Supplementary Table S1) and a monotonic pattern of increased levels of all terpenoids at all stages of incubation (Supplementary Fig. S1). We detected a clear and consistent pattern of increasingly higher levels of benzaldehyde in samples undergoing the *Rpv12*-mediated defence compared to controls during the entire course of the defence response (Fig. 4). We previously proposed benzaldehyde, a promoter of SA-mediated defence^44^, as a biomarker for the defence response in *Rpv3*-mediated resistance because this compound markedly increased at 48 and 96 hpi^20^. In *Rpv12*-mediated ETI, the rapid accumulation of this biomarker from 12 to 96 hpi suggests a more prompt and prolonged activation of the defence response compared to *Rpv3*-mediated ETI.

In order to identify the metabolites that better explain the differences between inoculated samples and controls, we selected 88 metabolites that showed an absolute value of the t statistic greater than 3 (|t|> 3) in one time point at least (Supplementary Fig. S2a, Supplementary Table S2). We summarized this information in the network represented in Supplementary Fig. S2b. We identified the largest number of nodes in the network at 48 hpi, indicating that the effects of metabolic reprogramming in samples undergoing *Rpv12*-mediated defence were maximal between 24 and 48 hpi and these changes involved mainly secondary metabolites.

### Key metabolite changes in secondary metabolism

**Figure 5:**
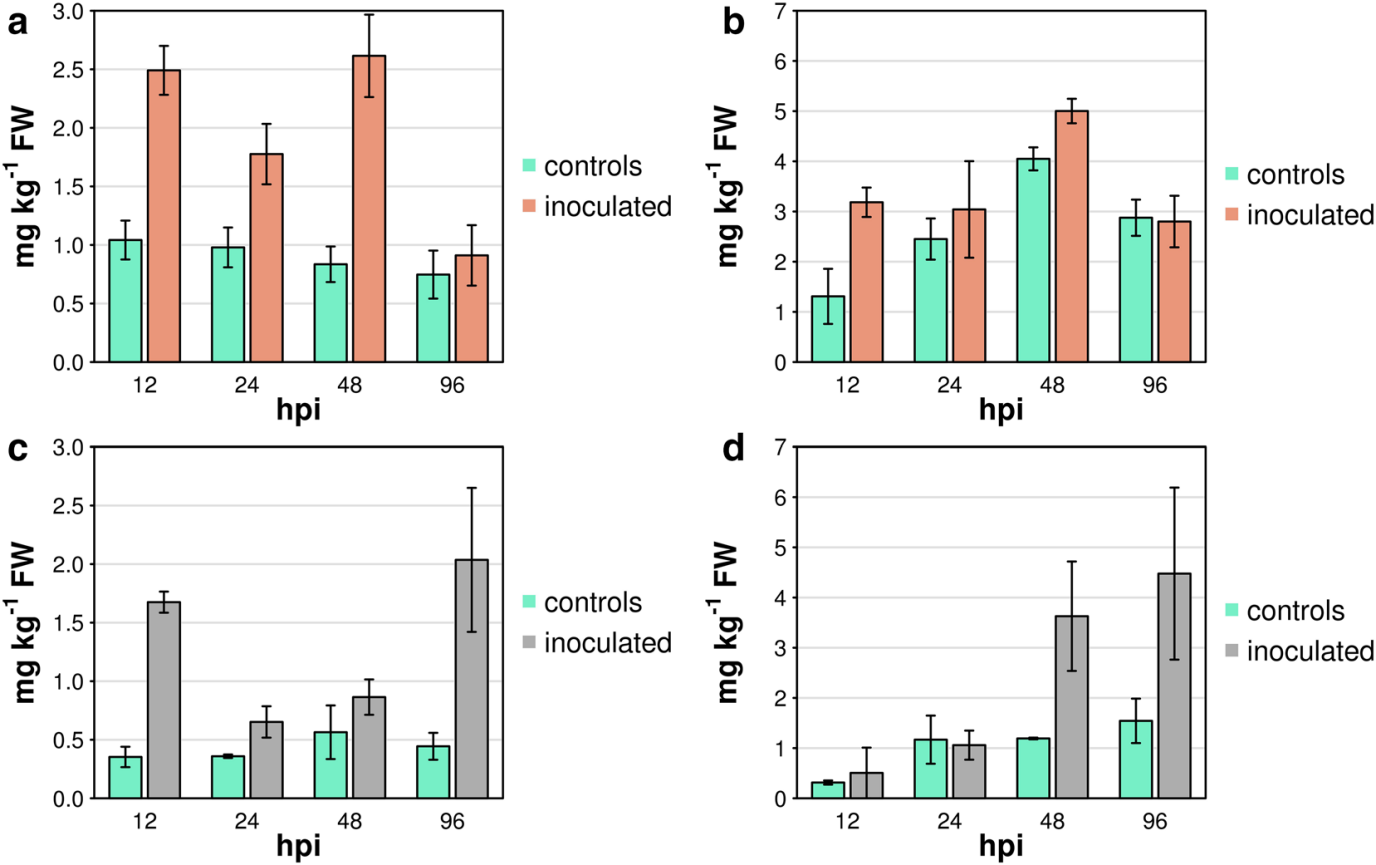
Concentration of *trans*-resveratrol (**a**,**c**) and ε-viniferin (**b**,**d**) in samples undergoing either (**a**,**b**) *Rpv12*- or (**c**,**d**) *Rpv3*-mediated defence response (inoculated) and controls. Graphs in (**c**,**d**) were plotted using raw data obtained from our previous paper^20^.

It has been previously shown that *Rpv3*-mediated defence is characterised by the two following early events: PCD occurs in the mesophyll cells underneath the infected stomata and non-toxic stilbenes, mainly *trans*-resveratrol, accumulate in leaves undergoing PCD. Elevated *trans*-resveratrol synthesis and PCD are operated between 12 and 24 hpi and their outcomes become effective, visible, and measurable between 24 and 48 hpi^12,20,23^. The conversion of non–toxic *trans*-resveratrol into fungi–toxic derivatives, mainly ε-viniferin, reached a peak between 48 and 96 hpi^20,23^. In the current experiment, the amount of *trans*-resveratrol was consistently two to threefold higher from 12 to 48 hpi in leaves undergoing *Rpv12*-mediated resistance than in controls (Fig. 5a), whereas this increase occurred more transiently from 12 to 24 hpi in our previous experiment with *Rpv3*-mediated resistance (Fig. 5c). As a result of this, the concentration of *trans*-resveratrol stayed within an higher range of variation (between 1.8 and 3.1 mg kg^−1^ of fresh weight (FW)) during the first 48 hpi in leaves undergoing *Rpv12*-mediated resistance, whereas the concentration remained within a lower range (between 0.7 and 1.7 mg kg^−1^ of FW) in *Rpv3*-mediated resistance^20^. This strong increase of *trans*-resveratrol in *Rpv12*-mediated resistance compared to controls was not paralleled or followed by a commensurate increase in ε-viniferin, which originates from oxidative dimerization of *trans*-resveratrol. The concentration of ε-viniferin was higher in inoculated samples undergoing *Rpv12*-mediated resistance than in controls only at 12 hpi (Fig. 5b), whereas it markedly increased at 48 and 96 hpi in *Rpv3*-mediated resistance (Fig. 5d).

### Impact of the defence response on the leaf transcriptome and global changes in metabolic pathways

**Figure 6:**
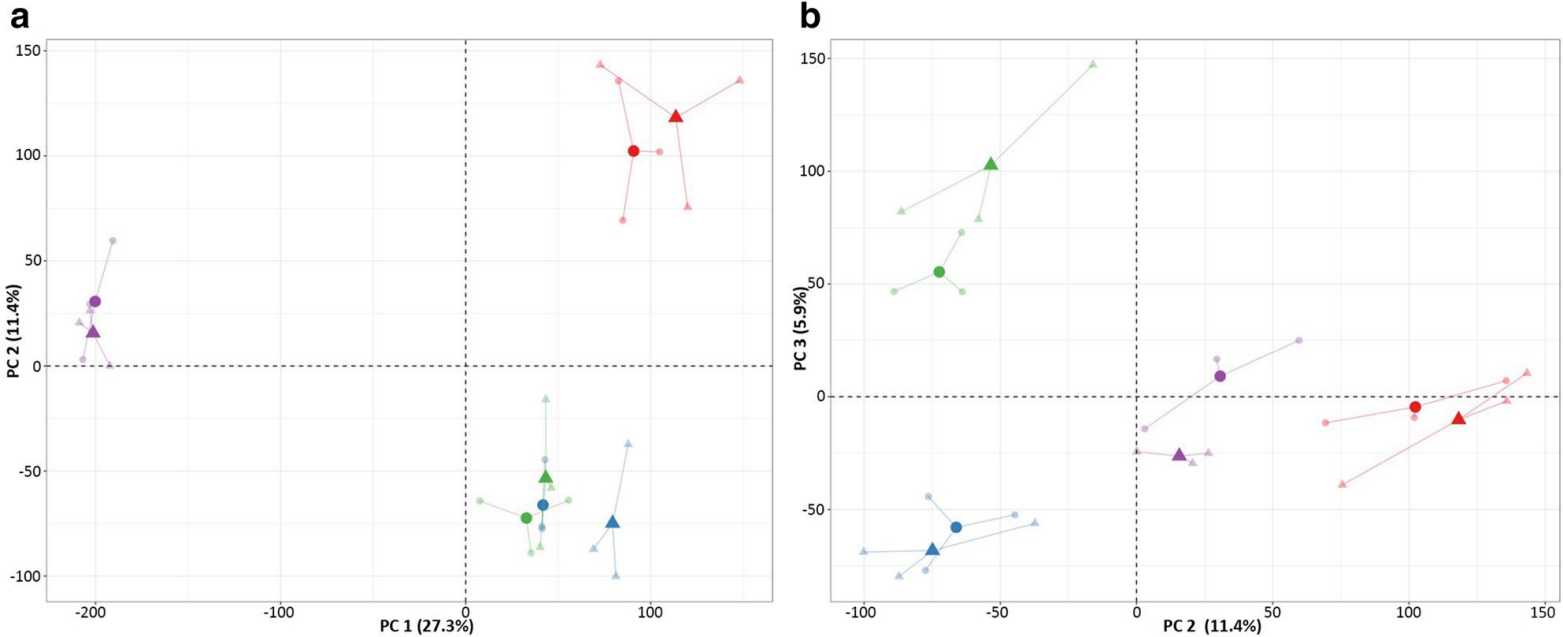
PCA of transcript data in leaves undergoing *Rpv12*-mediated defence (inoculated black filled circle) and controls (black filled triangle). Three biological replicates are reported as small symbols (black filled circle, black filled triangle) and are linked to their mean values (large symbols, black filled circle, black filled triangle) by connectors (–) for the first and second component in (**a**) and the second and third component in (**b**). Different colours represent different time points: red filled circle, red filled triangle 12 hpi; blue filled circle, blue filled triangle 24 hpi; green filled circle, green filled triangle 48 hpi; violet filled circle, violet filled triangle 96 hpi.

**Figure 7:**
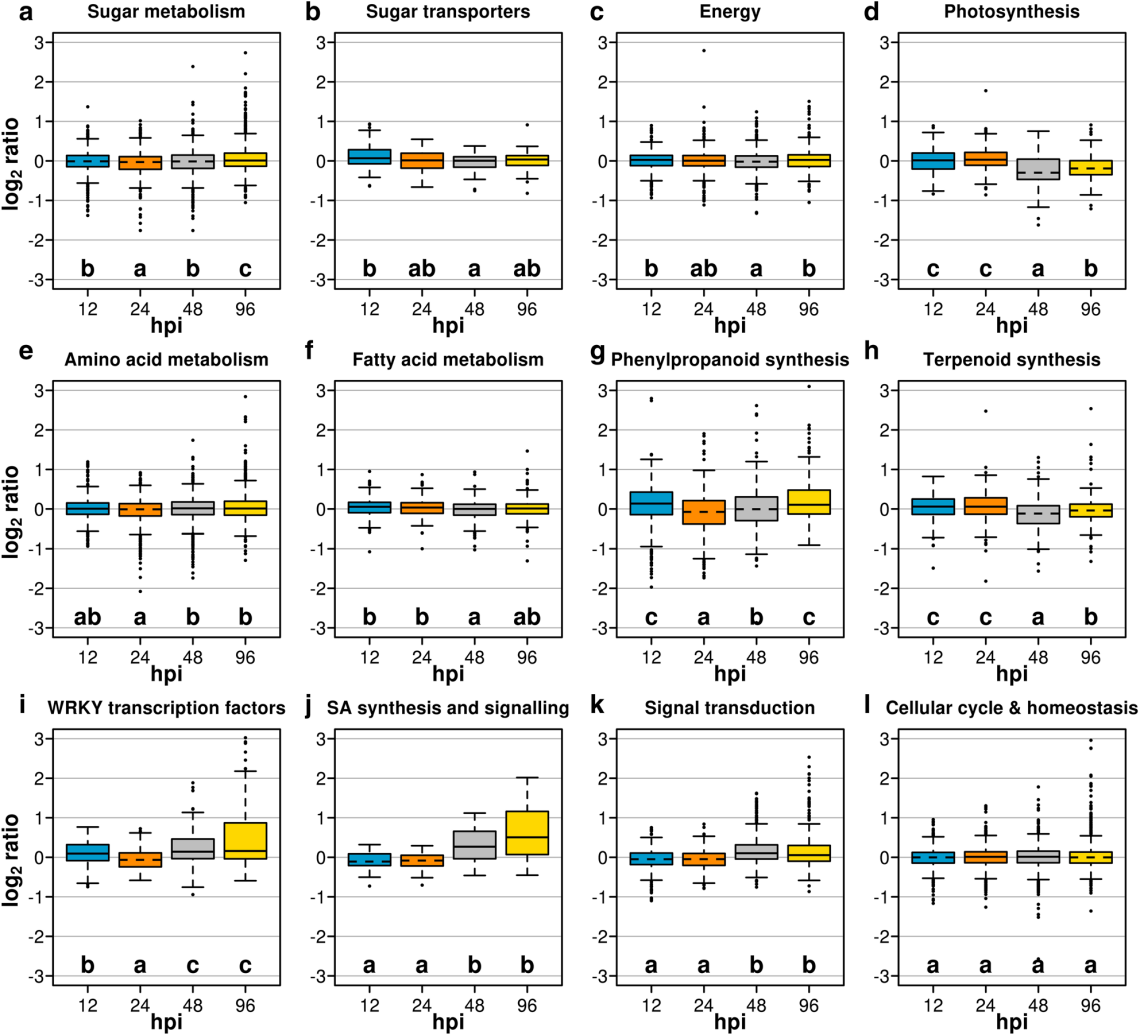
Global differences of gene expression in leaves undergoing *Rpv12*-mediated defence (inoculated) and controls. Genes were grouped by metabolic pathways (**a**–**l**). Box–plot distributions of FPKM log_2_ ratios at four stages of incubation. Each letter identifies distributions that are not significantly different using a Wilcoxon–Mann–Whitney test (*P* < 0.05). Alphabetical order indicates increasing median values. The line pointing to the median value in each box is plotted as a solid line for positive median values and as a dotted line for negative median values.

Figure 6 illustrates a lower-dimensional picture of the distribution of samples undergoing *Rpv12*-mediated defence response and controls during the course of the experiment based on a PCA using gene expression values for all transcribed genes. Replicates of samples undergoing *Rpv12*-mediated defence responses at 24 hpi grouped separately from replicated controls along the first component (PC1, Fig. 6a). Replicates of samples undergoing *Rpv12*-mediated defence responses grouped separately from replicated controls at 48 and 96 hpi along the third component (PC3, Fig. 6b). Figure 7 illustrates the distributions of FPKM log_2_ ratios between samples undergoing *Rpv12*-mediated ETI and controls at each stage of incubation for 12 groups of genes sorted by gene ontology (GO) category and/or metabolic pathway. Genes that are involved in sugar metabolism and generation of energy showed small global shifts in expression between samples undergoing *Rpv12*-mediated ETI and controls. The distributions of FPKM log_2_ ratios for sugar metabolism genes (Fig. 7a) were shifted towards negative median values from 12 to 48 hpi and were significantly shifted towards higher values and with a positive median value only at 96 hpi, suggesting that sugar metabolism pathways were slightly modulated at early stages in samples undergoing *Rpv12*-mediated ETI response. However, categories including genes that encode sugar transporters (Fig. 7b) and are involved in fatty acid metabolism (Fig. 7f) showed the most remarkable global shift in FPKM log_2_ ratios towards higher and positive values at 12 and 24 hpi. This is consistent with the notion that the activation of sugar signaling and fatty acid modulators of signal transduction pathways relies on mobilization and deployment of existing resources at the initial stages of the ETI response. These metabolic activities were sustained by the activation of pathways involved in generation of energy as we observed slight global shifts of distributions of FPKM log_2_ ratios for genes assigned to energy-related gene categories towards positive median values at 12, 24, and 96 hpi (Fig. 7c). Although the ETI response seems to require substantial amounts of carbon and energy, the global expression of genes involved in photosynthesis (Fig. 7d) suggested that these resources were not contributed by an activation of the photosynthetic pathway that was on the contrary strongly reduced at 48 and 96 hpi. We observed in samples undergoing *Rpv12*-mediated ETI two distinct temporal patterns of elevated expression of the gene family encoding WRKY transcription factors (Fig. 7i). A rapid and transient increase was detected at 12 hpi and was followed by a more sustained increase from 48 to 96 hpi. WRKY transcription factors are known to activate defence response in a number of ways through the up-regulation of genes involved in the phenylpropanoid pathway and in SA synthesis and signalling. Indeed, we found that structural genes of the phenylpropanoid and downstream pathways—including those required for stilbene synthesis—showed global shifts in the distributions of FPKM log_2_ ratios towards positive median values at 12 and 96 hpi (Fig. 7g). This observation recalls the notion that compounds in this class activate early signalling pathways and, at later stages of the incompatible interaction, sustain the production of stilbenes. We also observed statistically significant shifts towards higher FPKM log_2_ ratios with positive median values in the distribution for genes involved in SA synthesis and signalling at 48 and 96 hpi (Fig. 7j), concomitant with similar increases in the expression of genes involved in calcium and mitogen activated protein (MAP) kinase signalling (Fig. 7k). We finally used the set of genes assigned to the GO categories of cell cycle and cell homeostasis that are not expected to be recruited in defence responses as a negative control for this analysis. We indeed observed that these GO categories showed no statistically significant difference in their global distributions of FPKM log_2_ ratios between stages and their median log_2_ ratios pointed to zero at each stage of incubation (Fig. 7l).

### Statistically significant and major differences in individual gene expression

We then defined a set of differentially expressed genes (DEGs) between inoculated samples and controls by selecting genes with an absolute value of log_2_ Fold Change (FC) in expression greater than 1 and adjusted *p* value *P* < 0.05. We found 431 DEGs including alternative transcripts with differential expression for 35 genes (Supplementary Table S3). We validated by quantitative PCR (qPCR) a sample of DEGs representative of different GOs and found consistency both in the log_2_ ratios (Supplementary Fig. S3) and in the statistical significance of the difference (Supplementary Table S4) between samples undergoing *Rpv12*-mediated ETI and controls. Only two DEGs were identified at 12 hpi, which were both up-regulated in samples undergoing *Rpv12*-mediated defence responses. These two genes are *VIT_207s0129g00660* encoding an Indole-3-acetic acid (IAA)-amido synthetase (IAA) and *VIT_209s0002g06890* encoding a blue copper protein. IAA-amido synthetases, also known as GH3 proteins, dampen free-IAA levels by catalysing the conjugation of IAA to amino acids. GH3-overexpressing plants in Arabidopsis and rice showed enhanced resistance to *Pseudomonas syringae*^45^ and *Magnaporthe oryzae*^46^, respectively, and it is generally assumed that suppression of IAA signalling is an integral part of SA-mediated resistance against biotrophic pathogens^47,48^. The second up-regulated gene (*VIT_209s0002g06890*) showed elevated expression levels also at 24 and 48 hpi. We found 70 DEGs at 24 hpi (17 up-regulated; 53 down-regulated in samples undergoing *Rpv12*-mediated defence responses); 50 DEGs at 48 hpi (41 up-regulated; 9 down-regulated in samples undergoing *Rpv12*-mediated defence responses) and 340 genes at 96 hpi (326 up-regulated; 14 down-regulated in samples undergoing *Rpv12*-mediated defence responses). The expression of a fraction of genes was statistically different between samples undergoing *Rpv12*-mediated defence responses and controls at more than one stage of incubation as reported in the Venn diagram of Supplementary Fig. S4. We focused our attention on the top 100 most significantly enriched GO categories for biological processes represented in the list of DEGs and the 20 GO categories with lowest *p* values are shown in Supplementary Table S5. At 48 and 96 hpi, we found up-regulation of DEGs belonging to the “plant hypersensitive response” (GO:0009626). We also found up-regulation for DEGs belonging to GO categories such as the “salicylic acid biosynthetic process” (GO:0009697), “systemic acquired resistance, salicylic acid mediated signalling pathway” (GO:0009862) and “response to salicylic acid stimulus” (GO:0009751). It was worth noting that at the same stages of incubation we found a significant up-regulation of the gene *VIT_208s0058g01390* encoding a WRKY70 transcription factor that acts in Arabidopsis as an activator of SA-induced PR genes and is required for *RPP4*-mediated disease resistance against *Hyaloperonospora parasitica*^49^. It is known that resistance genes activate ETI responses through the SA-dependent signalling pathway and through signalling cascades that amplify the spectra of defence responses. Indeed, we found up-regulation of DEGs belonging to the “MAP kinase cascade” category (GO:0000165). For instance, the gene *VIT_209s0002g04560* encoding a calmodulin-binding protein was significantly up-regulated at 48 and 96 hpi in samples undergoing *Rpv12*-mediated defence responses. Calmodulin-binding proteins play crucial roles in cellular signalling cascades through the regulation of numerous target proteins^50^. We also observed up-regulation of the gene *VIT_203s0088g00910* encoding a class 1 pathogenesis related protein (PR-1) at 24, 48 and 96 hpi and of the gene *VIT_202s0025g04330* encoding a thaumatin-like class 5 protein (TLP or PR-5) at 48 and 96 hpi in samples undergoing *Rpv12*-mediated defence responses (Supplementary Table S4). At 24 and 48 hpi we also found up-regulation of DEGs *VIT_208s0007g06040* and *VIT_208s0007g06060* encoding beta-glucanases that operate cell wall lysis of invading pathogens as a part of the inducible defence responses. DEGs with predicted functions related to amino acid, carbohydrate and lipid metabolism were up-regulated at 96 hpi together with genes belonging to secondary metabolism pathways within the GOs “*coumarin biosynthetic process*” (GO:0009805), and “*stilbene biosynthetic process*” (GO:0009811). At 96 dpi, we also found significant down-regulation of individual genes involved in photosynthesis and photosynthetic electron transport chain processes, such as *VIT_210s0003g02900, VIT_217s0000g06350, VIT_210s0003g02890* encoding different chlorophyll a-b binding proteins.

### MCIA of metabolites and transcripts

**Figure 8:**
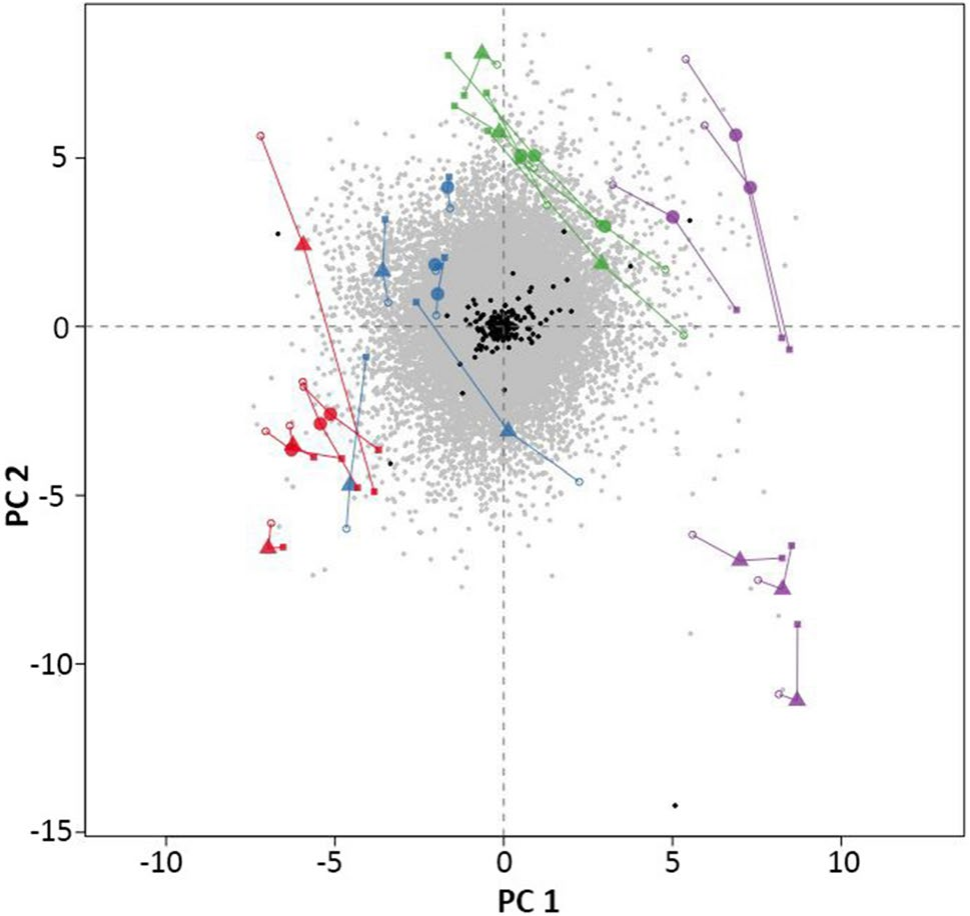
Multiple Co-Inertia Analysis of two-omics datasets including all transcript levels (grey filled square) and metabolite concentrations (black filled circle). The two axes report the first two principal components (PC). Small coloured symbols summarise transcript or metabolite data separately for each replicated sample and stage (red filled square, red open circle 12 hpi; blue filled square, blue open circle 24 hpi; green filled square, green open circle 48 hpi; violet filled square, violet open circle 96 hpi, respectively) and are linked by connectors (–) to their integrated value for samples undergoing *Rpv12*-mediated defence (red filled circle; blue filled circle; green filled circle; violet filled circle) and controls (red filled triangle; blue filled triangle; green filled triangle; violet filled triangle).

We used Multiple Co-Inertia Analysis (MCIA), a method developed by Meng et al.^51^, to identify co-relationships between the two-omics datasets using a multivariate dimension reduction approach. MCIA rescales the values from heterogeneous datasets using a covariance criterion and projects simultaneously multiple datasets onto the same plane. The results of MCIA analysis are reported in Fig. 8. Individual transcripts and metabolites are represented by grey filled squares and black filled dots, respectively, regardless of the sample type, of the stage of incubation and of the replicate. The colored dots, which are differentiated by the stage of incubation, recapitulate the global distribution of each omics dataset on the same bidimensional space, sorted by sample type and replicate. Large colored triangles and circles recapitulate the integration of the two-omics datasets, sorted by sample type, stage of incubation and replicate. The length of the connectors between large symbols and the corresponding small coloured dots are inversely correlated with the extent to which the two-omics datasets are coherent in the separation by sample type, stage of incubation and replicate on the bidimensional space. The MCIA plot showed that the strongest separation between samples undergoing *Rpv12*-mediated defence responses and controls occurred at 96 hpi.

## Discussion

In grapevine leaf discs that are operating *Rpv12*-mediated resistance against the infection of *P. viticola*, the host tissues are characterised by wider and faster metabolome changes than those observed in a similar experiment performed on leaf discs that were operating *Rvp3*-mediated resistance^20^. Using the same analytical and statistical methods and the same cut-off thresholds, we identified a higher number of metabolites (88 versus 53) that were significantly modulated over a 96 h period after the inoculation with the pathogen. We also found that substantial metabolite changes occurred earlier in *Rpv12*-mediated resistance compared to *Rpv3*-mediated resistance with the highest number of modulated metabolites at 48 hpi instead of 96 hpi^20^. Although we paid particular attention to the choice of an appropriate *Rpv12*-carrying genotype in order to minimise the confounding effects of the genetic background, we cannot exclude that part of the faster and stronger defence responses of the *Rpv12*-carrier compared to the *Rpv3*-carrier was due to more effective signal transduction components downstream of the R-gene in the genotype under study. We indeed observed substantial variation in field resistance against natural infections of *P. viticola* among dozens of grape varieties that all carry the same *Rpv3-1* resistance haplotype in an ample variation of genetic backgrounds^52^. While for choosing the *Rpv3*-carrier^20^ we had background information available to select an average variety, such as ‘Bianca’, as a representative of a known distribution^52^, similar data are still not available for a substantial number of *Rpv12*-carriers, due to a more recent origin of this lineage. While *Rpv3*-carrying varieties had been introduced in cultivation since the late 1800’s and since then used for further breeding, it was only in 1960 that Koleda and Tamássy backcrossed *V. amurensis* × *V. vinifera* hybrids to generate the first two resistant varieties (‘Kunleany’ and ‘Kunbarat’^53^) carrying a new resistance that later has become known as *Rpv12*-dependent^21^. It took another 15 years after Koleda and Tamássy, before Cindrić and Korać had the intuition that those two varieties could become excellent progenitors for a new lineage of resistant varieties, performing in 1977 the backcross of ‘Kunbarat’ that has led to origin of the individual SK77-4/5^54^, a parent of the individual of this study. It is therefore possible that we used by chance, among the few available, an *Rpv12*-carrier with a particularly favourable genetic background, although several reports converge on a consensus that, based on OIV452 scores, *Rpv12*-dependent resistance is stronger across varieties than *Rpv3*-dependent resistance^55,56^. Bove and Rossi^57^ also showed that three varieties operating *Rpv12*-dependent defence clustered together and separately from varieties carrying other *Rpv* genes for five defence components (i.e. proportion of inoculated sites that develop disease symptoms, length of the latent period, number of waves of sporangial release during the infectious period upon extended incubation of sporulating lesions, sporangial density per lesion, infectivity of the released sporangia), suggesting that the outcome of the defence response is still more dependent on the type of R-gene than on the genetic background of the variety. Despite the inherent limitations of this kind of comparative experiments, we observed that changes leading to a common metabolic reprogramming necessary for triggering and operating cell death at the infection sites and for SA-dependent synthesis of PR-proteins have occurred in *Rpv12*-mediated resistance with similar timing and in the same direction as observed for *Rpv3*-mediated resistance.

While the causal factor that triggers *Rpv3*-mediated HR has been identified in the gene products of two nearby TIR-NB-LRRs^58^, the causal gene that would explain the observed Mendelian inheritance of *Rpv12*-mediated resistance has not been isolated yet^21^. The sequence of the reference grapevine genome that has been assembled from a DM sensitive genotype predicts the presence of allelic forms of CC-NB-LRR genes in the *Rpv12* locus^21,59^. Although the *Rpv12*-resistance haplotype has yet to be sequenced, the *Rpv12*-mediated response analysed in this paper shows the hallmarks of a ROS- and SA-dependent HR triggered by a NB-LRR gene product. Here, we provided evidence that gene expression profiling and changes in transcription levels of gene categories grouped by metabolic pathways lend support to the observed metabolite changes over a period of 96 hpi and highlight striking similarities with the canonical model of ETI-mediated defence responses to biotrophic pathogens in plants^60^. Indeed, cumulative evidence from metabolomic and transcriptomic data suggests that the *Rpv12*-mediated resistance relies on a rapid activation of a broad set of inducible responses that take place within 12 hpi. At 12 hpi, we detected elevated expression of gene categories encoding sugar transporters, which suggest a mobilisation of existing carbon resources, and an increase of sugar concentration. Sugar sensing and sugar-induced signalling have been shown to activate pathways leading to the synthesis of PR-proteins, to elicit PCD^61^ and to induce a metabolic swift from source to sink through a negative feedback loop^62^. At 12 hpi, samples undergoing *Rpv12*-mediated ETI also showed elevated concentration of linolenic acid that is reported to directly activate NADPH-oxidase^41^, an enzyme required for oxidative burst. An increase in linolenic acid has also been observed at the early stages of the *Rpv3*-mediated defence response in ‘Regent’^40^. Elevated levels of *trans*-resveratrol were observed at 12 hpi in both *Rpv12*- and *Rpv3*-mediated defence responses. In contrast with metabolite and transcript analyses of *Rpv3*-mediated^20,23^ and of *Rpv10*-mediated defence^35^, we did not find statistically significant differences between samples undergoing *Rpv12*-mediated defence response and controls in the expression of the gene *VIT_212s0028g01840*, which encodes a peroxidase possibly involved in the dimerization of *trans*-resveratrol into the fungi-toxic ε-viniferin. Nor did we find evidence for a significant increase of ε-viniferin concomitant with or subsequent to the increase of *trans*-resveratrol in samples undergoing *Rpv12*-mediated defence response. Although the two roles are not mutually exclusive, these lines of evidence in *Rpv12*-mediated resistance point to a primary role of *trans*-resveratrol as a signaling molecule in ROS formation and initiation of programmed cell death, as proposed by^63^, rather than as a precursor of toxic stilbenes, as it occurs in *Rpv3*- and in *Rpv10*-mediated defence. An augmented and prolonged accumulation of resveratrol, without a substantial conversion into ε-viniferin, has also been observed in otherwise sensitive grapevines after they were administered a specific sulphated derivative of laminarin that primed H_2_O_2_ production and suppressed the development of *P. viticola* in inoculated leaf discs^64^.

This ample set of early changes that had already occurred by 12 hpi in samples undergoing *Rpv12*-mediated defence suggests that the metabolism is timely reconfigured shortly after pathogen perception and the entire defence machinery comes immediately into operation as a part of a crisis management program. However, the full deployment of the activated defence weaponry remains on standby until a point of no return that is surpassed when ROS and SA synergistically potentiate the response and drive cells into an auto-toxic and energetically costly process. At 24 hpi, we observed strong reduction in 18:1 fatty acids that suggests initiation of ROS production, as it occurs in the *Rpv3*-mediated defence response with a timing that coincides in that case with the execution of PCD^23^. In Arabidopsis, it is reported that lower levels of 18:1 fatty acids directly stabilise the NITRIC OXIDE ASSOCIATED1 enzyme, increasing nitric oxide concentration^65^, a signalling gaseous radical that potentiates ROS-dependent PCD and up-regulates gene expression in the phenylpropanoid and SA pathways. It is possible that we only detected early transcriptional and metabolic signals associated with the onset of PCD at 24 hpi. Light exclusion—an experimental condition imposed for minimising the effect of light-dependent circadian fluctuations on the transcriptome at different sampling stages—might have prevented us from observing the occurrence and consequences of light-dependent oxidation, including lipid peroxidation^40^, and ROS production that are known to regulate the strength of PCD-dependent HR. However, we observed a similar trend of changes in fatty acid composition in response to *Rpv12*-mediated defence response as that reported by^40^ in *Rpv3*-mediated defence response in ‘Regent’, which did not occur in sensitive host controls^40^. These authors reported a transient and slight increase of oleic, linoleic and linolenic acids at the earliest stages of the defence response (6 hpi), followed by a rapid decrease of their concentrations at 12 hpi^40^. We observed a delay in this trend, with slight increases at 12 hpi and decreases at 24 hpi, but the drop at 24 hpi was much more pronounced and consistent with what we previously observed in *Rpv3*-mediated defence response in ‘Bianca’^20^.

We observed at 48 hpi full deployment of important components of the defence weaponry, such as SA-signalling, calcium and MAP kinase signal transduction, PR gene expression, and strong repression of photosynthesis that marked the complete source-to-sink reconfiguration of the metabolism to shuttle resources into energy-generating pathways and to prioritize the synthesis of defence-related compounds. The hypothesis of a central role of SA-signalling in eliciting some components of the *Rpv12*-mediated response was corroborated by the observation of a general up-regulation of the WRKY family of transcription factors. Noteworthy was the up-regulation of grapevine homologs of WRKY28 that binds to the promoter of isochorismate synthase^66^, the rate-limiting enzyme in SA biosynthetic, and of WRKY70, a component of gene-for-gene resistance against the oomycete *Hyaloperonospora parasitica* in Arabidopsis that causes induction of genes encoding PR-1, PR-2, and PR-5 proteins^49,67^. The grapevine VvWRKY28 has also been proposed as a positional candidate residing in a QTL confidence interval that explained 10.8% of the phenotypic variation for *cis*-resveratrol content in a segregating population^68^. In the same work, some stilbenoids showed a general trend of augmented concentration in individuals with higher OIV-452 scores and OIV-452 scores were higher in full-sibling individuals that carried an *Rpv3*-*3* haplotype than in those who did not. However, no significant QTL for stilbenoid concentration was found to colocalise with the QTL for OIV-452 scores, which corresponded to the *Rpv3* locus and explained 19.7% of the observed variation in *P. viticola* resistance^68^. Another transcription factor, VvWRKY33, was found by^69^ to be induced in ‘Regent’ during the *Rpv3*-mediated response from 2 to 120 hpi, with highest differences in expression levels compared to mock-inoculated controls between 4 and 10 hpi and correlation with increased expression of a pathogenesis related protein 10.1 (PR-10.1) gene. *VIT_208s0058g00690* is the most similar reference gene for the WRKY33 sequence isolated from ‘Regent’ (GenBank accession no. KF800706.1^69^). We found elevated levels of *VIT_208s0058g00690* expression at 48 and 96 hpi in leaf discs undergoing the *Rpv12*-mediated response compared to controls (log_2_ Fold Change 0.70 and 0.88, respectively for the primary transcripts), although these difference remained below the threshold of statistical significance. In a similar way, we found a trend of augmented levels, yet not statistically significant, of three PR-10.1 gene copies (*VIT_05s0077g01530*, *VIT_05s0077g01580*, *VIT_05s0077g01560*) within a tandemly arrayed gene cluster at 48 hpi (log_2_ Fold Change 0.19, 0.16, 0.16, respectively) and 96 hpi (log_2_ Fold Change 0.19, 0.23, 0.23, respectively) in leaf discs undergoing the *Rpv12*-mediated response. In the *Rpv12*-mediated response, we vice versa found statistically significant induction of a thaumatin-like protein gene similar to a *V. amurensis* ortholog that impaired mycelial growth, causing hyphal malformation, in transgenic lines of sensitive controls^70^. An increasing number of yet genetically uncharacterized sources of *P. viticola* resistance have emerged in the past years from Asian species, which include *V. pseudoreticulata*^71^ and *Vitis quinquangularis*^72^. Further studies will be needed to clarify if these novel downy mildew resistances share the same type of ETI and follow the same lines of defence reactions as those observed in *Rpv12*-mediated resistance.

## Materials and methods

### Plant material and artificial inoculation

Dormant canes for the experiment were obtained from mother plants held in a germplasm repository in the South Tyrol region, Italy. Two bud cuttings (*n* = 45) were rooted in potted soil and forced to sprout in isolation within a greenhouse free from sources of inoculum. A single shoot was raised from each rooted cutting until the stage of 12 expanded leaves. Water was supplied by capillary action by placing the pots, containing the rooted cuttings, onto a layer of capillary matting that was kept constantly wet. We then sorted the plants into three homogenous groups. Each group of plants (*n* = 15) formed a biological replicate. At the time of the inoculation the plants were healthy, with no symptoms of any foliar disease. In parallel, shoots were raised from three cuttings of the variety ‘Pinot Noir’ in order to prepare sensitive controls. The three distal fully expanded leaves were detached from all plants of a biological replicate, bulked, and rinsed with ultrapure water. Discs were excised from each leaf lamina using a 1.1-cm diameter cork borer and plated randomly with the abaxial surface upside on wet paper in Petri dishes, collectively representing the biological replicate. Leaf discs were acclimated at 21 °C in the dark for 12 h in order to dispel wounding-induced jasmonic acid signalling^73^. Petri dishes from each biological replicate were divided randomly into two groups, one used for pathogen inoculation and one used for mock-inoculation, hereafter referred to as controls. Inoculated samples were sprayed with a *P. viticola* suspension of 10^6^ sporangia ml^−1^, while controls were sprayed with ultrapure water. *P. viticola* was originally collected as a natural mixture of sporangia from diverse *V. vinifera* cultivars in an unsprayed vineyard in San Michele all’Adige, Northern Italy. Sporangia were stored at − 20 °C prior to use for the experiment of this study and for the experiment reported in^20^, which was conducted two weeks earlier during the same growing season. The inoculum was propagated on plants of *V. vinifera* ‘Pinot Gris’ grown in potted soil in a greenhouse by spraying a 5 × 10^5^ ml^−1^ suspension of the frozen sporangia in distilled water. Plants were maintained at > 90% relative humidity at 21 °C until sporulation. Fresh sporangia were collected from ‘Pinot Gris’ plants by soaking sporulating leaves in refrigerated (4 °C) distilled water. The suspension was diluted to the final concentration using a Burker chamber and sprayed immediately on the experimental leaf discs. All Petri dishes were incubated at 21 °C in the dark until sampling. Samples were collected at 12, 24, 48 and 96 hpi and immediately frozen in liquid nitrogen. A set of five Petri dishes was available per sampling stage, per treatment and per biological replicate. Four Petri dishes in each set collectively containing a minimum of 100 leaf discs were used for metabolomic analyses by pooling and grinding together all discs of the biological replicate. The remaining Petri dish of each set containing a minimum of 25 leaf discs was used for transcriptomic analyses by sampling two discs per time point that were pooled and ground together for RNA extraction. The remaining leaf discs were incubated until sporulation to confirm *P. viticola* infection on inoculated samples and to exclude *P. viticola* contamination prior to sample collection on mock-inoculated controls. Sporulation was not observed on inoculated *Rpv12*-resistant leaf discs during the course of sampling and until 120 hpi. The resistance phenotype scored a value of 9 on the OIV452 descriptor scale^12^, due to concomitant absence of sporulation and presence of barely visible necrotic spots. Sporulation was already visible at the last stage of sampling (96 hpi) in inoculated control leaf discs of the sensitive ‘Pinot Noir’ and was confirmed after the conclusion of the experiment at 120 hpi. Sporulation was not observed on mock-inoculated control leaf discs of ‘Pinot Noir’ until 120 hpi. Gene expression analyses were performed at the genomics facility of the Fondazione Edmund Mach. Three biological replicates per treatment per time point were used for metabolite and gene expression analyses.

### Metabolite analysis

Primary metabolites were determined following the methodology published by Chitarrini and coworkers^20^. GC/MS analysis was performed by injecting 1 µL of derivatised extract into a Trace GC Ultra instrument combined with a TSQ Quantum GC mass spectrometer (Thermo Electron Corporation, MA). Compounds were separated using an RXI-5-Sil MS w/Integra-Guard column. Data acquisition was run in full scan mode from 50 to 700 m/z. Data were processed using the XCALIBUR 2.2 software.

Lipids were determined using the method developed by Della Corte et al.^74^. Chromatographic analysis was carried out using a UHPLC Dionex 3000 instrument (Thermo Fischer Scientific, DE) combined with an API 5500 triple quadrupole mass spectrometer (Applied Biosystems/MDS Sciex, CA). Compounds were separated using an RP Ascentis Express column.

Phenols were determined with slight modifications of the method developed by Vrhovsek et al.^75^. Chromatographic analysis was carried out using a Waters Acquity UPLC instrument combined with a Waters Xevo triple quadrupole mass spectrometer detector. Compounds were separated using a Waters Acquity HSS T3 column. Data processing was performed using Waters MassLynx V4.1 software.

Primary metabolites, lipids, and phenols. Compounds were identified based on their reference standard, retention time and qualifier and quantifier ion. Compounds were quantified using linear calibration curves and expressed as mg kg^−1^ of FW.

Volatile compounds were extracted with slight modifications of the methods developed by Matarese et al.^76^ and Salvagnin et al.^77^ as described by Chitarrini et al.^20^. Compounds were captured from the headspace using a Supelco 2-cm DVB/CAR/PDMS 50/30 μm fibre (Supelco, PA). Chromatographic analysis was carried out using a Trace GC Ultra gas chromatograph combined with a Quantum XLS mass spectrometer (Thermo Scientific, Electron Corporation, MA). Compounds were separated using a Stabilwax-DA column (Restek Corporation, PA). Data were processed using the XCALIBUR 2.2 software. Identification of volatile compounds was carried out by injecting pure reference standards when available or comparing retention index and mass spectra using the NIST MS Search 2.0 database. Results were expressed in µg kg^−1^ of FW with semi-quantification using 1-heptanol as internal standard.

### RNA extraction and gene expression analysis

Approximately, 100 mg of leaf discs were ground to powder in liquid nitrogen. RNA was isolated according to the manufacturer’s instructions using the Spectrum Plant Total RNA Kit (Sigma-Aldrich, DE). Total RNA quality was checked on RNA ScreenTape using an Agilent 2200 TapeStation (Agilent Technologies, CA).

### RNA-Seq and gene expression analysis

cDNA libraries were constructed using 1 µg of total RNA and the KAPA Stranded mRNA-Seq Kit (Kapa Biosystems, MA). Each library was barcoded using the SeqCap Adapter Kit A and B (NimbleGen, Roche, CA) and the target range of fragment sizes (250–280 bp) was confirmed on High Sensitivity D1000 ScreenTape using the Tapestation 2200 (Agilent Technologies, CA,). All the libraries were quantified with a KAPA Library Quantification Kit (Kapa Biosystems, MA) using a LightCycler 480 (Roche, DE) and multiplexed in four equimolar pools. Each library pool was sequenced on an Illumina HiSeq 2500 sequencer generating 2 × 50 bp paired-end reads. An average of 54.4 million paired-end reads was generated per sample. Base calling and quality control were performed using the Illumina RTA sequence analysis pipeline. Reads were aligned to the grapevine PN40024 reference transcriptome^59,78^ using Bowtie2^79^, obtaining an average rate of read alignment of 84.7%.

### Use of RNA-Seq data for the description of the genetic makeup of the genotype under-study

We also used RNA-Seq reads from all samples and stages to perform allele-specific read alignment against a modified version of the grapevine genome reference that included the reference *Rpv3*-*PN40024* haplotype and the alternative resistance *Rpv3*-*1* haplotype and called variant sites using the procedure described in^58^. RNA reads revealed a substantial number of homozygous sites in transcribed genes of the *Rpv3*-*1* sequence, which allowed us to exclude that the genotype analysed in this study had inherited from its parent ‘Bianca’ the resistance haplotype at the *Rpv3* locus. To corroborate this analysis and to widen the description of the genetic constitution of the genotype under study, we realigned RNA-Seq reads from all samples and stages against the canonical grapevine genome reference^59^ with TopHat2 and called variants sites in transcribed genes with GATK. We filtered all SNPs present in a comprehensive diversity-panel of *vinifera* as described in^58^ and plotted the density of residual (non-*vinifera*) SNPs in 100-Kb genomic windows. We generated evidence that the introgression in the upper arm of chromosome 14 extends from the upper telomere down to a genomic window whose distal edge is located at the chromosomal coordinate chr14:11,683,740 (Supplementary Fig. S7), approximately 2 Mb downstream of the reported location of the *Rpv12* locus^21^. A closer inspection into the introgression around the *Rpv12* locus was conducted using available DNA sequencing reads of ‘Merlot Khorus’, a variety known to carry the *Rpv12* resistance haplotype, which derived from the backcross to *vinifera* of a full-sibling of the genotype under study (uncle–nephew relationship), and UD-21076^58^, which derived from the selfing of ‘Bianca’, the second parent of the genotype under study (Supplementary Fig. S8). We could extract evidence from the nearly complete sharing of non–*vinifera* SNPs between ‘Merlot Khorus’ and the genotype under study (Supplementary Fig. S8e) that they indeed share the same Asian introgression across a large part of the upper arm, including the *Rpv12* locus (Supplementary Fig. 8a,c–e). The resistance chromosome recombined with the *vinifera* counterpart at 11,7 Mbp in the megaspore mother cell of SK77–4/5 that formed the gamete transmitted to the genotype under study. We could also confirm that the homologous chromosome was donated to the genotype under study by ‘Bianca’, because this homolog shared with UD–21,076 exactly the same recombination break points at two introgressed regions (Supplementary Fig. S8b–e). One of these introgressions extended from 5.8 to 11 Mbp. As a result of this, the individual under study is heterozygous for an Asian (*Rpv12* resistance haplotype) and an American introgression across approximately 5 Mbp that also include the *Rpv12* locus (Supplementary Fig. 8c). Since we did not detect any significant QTL effect on *P. viticola* resistance on chromosome 14 in ‘Bianca’^12^, which is heterozygous for the American introgression and a *vinifera* haplotype across this locus, we infer that this particular haplotypic combination occurred by chance and was not a target of intentional selection in this individual. We also confirmed that the genotype under study carries two *vinifera* homologouos copies along the entirety of chromosomes 18 and 9 (Supplementary Fig. S7) and it did not show any signal of introgression in or around the reported locations of the *Rpv3* locus^58^ and the *Rpv10*^80^ locus on those chromosomes.

### qPCR validation

cDNA was generated using Invitrogen SuperScript IV VILO Master Mix (ThermoFisher Scientific, DE) and 1.5 µg of total RNA. A set of 9 DEGs representative of different GO categories were selected for primer design and validation (Supplementary Table S3). Quantitative PCR reactions were performed using a LightCycler480 thermocycler (Roche, CH) and a 2 × qPCRBIO SyGreen Kit (PCR Biosystems, UK) following the manufacturer’s instructions. Transcripts were quantified using the LC480 software (Roche, CH) and the 2^–∆∆CT^ method using the glyceraldehyde-3-phosphate dehydrogenase (GADPH) housekeeping gene VIT_17s0000g10430 for normalisation with the following primer sequences (forward 5′ → 3′ TTCTCGTTGAGGGCTATTCCA; reverse 5′ → 3′ CCACAGACTTCATCGGTGACA).

### Data analysis and graphical representation

Missing data points were replaced with a random value between zero and the limit of quantification of each metabolite. A log_10_-transformation of metabolite concentration was performed prior to statistical analysis^81^. The t-statistic was computed using the R package Stats^82^. Metabolite networks were generated using ggraph^83^. DEG analysis was performed with DeSeq2^84^. Gene enrichment analysis was performed using blast2GO^85^. Gene functional annotation was retrieved from VitisNet^86^, VitisCyc^87^, and V2.1 annotation^78^. A two-sided Wilcoxon–Mann–Whitney test was used for assessing significant differences between distributions of values. Multivariate data analysis was performed using the R package FactoMineR^88^ and the related graphs in Figs. 1 and 6 were generated using the R package Factoextra^89^. The R package omicade4^51^ was used to perform MCIA and to generate the plot reported in Fig. 8. Graphs in all other figures were generated with R (version 3.3)^82^. Metabolite concentrations of *Rpv3*-mediated defence response were obtained from raw data of our previous paper^20^.

## Supplementary information

Supplementary Figures.

Supplementary Table S1.

Supplementary Table S2.

Supplementary Table S3.

Supplementary Table S4.

Supplementary Table S5.

## Data Availability

RNA-Seq reads were deposited in the European Nucleotide Archive repository under the Unique Name Experiment ERX2989847. Metabolite data are provided as Supplementary Table S1. ‘Merlot Khorus’ FASTQ files of the reads aligning to chromosome 14 are publicly available at figshare under the https://doi.org/10.6084/m9.figshare.12240881.
