# Supplementary material for: Two-omics data revealed commonalities and differences between Rpv12- and Rpv3-mediated resistance in grapevine
Source: Sci Rep. 2020 Jul 22;10:12193. doi: 10.1038/s41598-020-69051-6 (PMC7376207; doi:10.1038/s41598-020-69051-6)
Supplement: Supplementary file 1 — Supplementary Figures. [file 41598_2020_69051_MOESM1_ESM.docx]

Two–omics data revealed commonalities and differences between Rpv12– and Rpv3–mediated resistance in grapevine

Giulia Chitarrini^1^, Samantha Riccadonna^1^, Luca Zulini^1^, Antonella Vecchione^1^, Marco Stefanini^1^, Simone Larger^1^, Massimo Pindo^1^, Alessandro Cestaro^1^, Pietro Franceschi^1^, Gabriele Magris^2,3^, Serena Foria^2^, Michele Morgante^2,3^, Gabriele Di Gaspero^3^*, Urska Vrhovsek^1^*.

^1^ Research and Innovation Centre, Fondazione Edmund Mach, via E. Mach 1, 38010 San Michele all’Adige, Italy

^2^ Department of Agricultural, Food, Environmental and Animal Sciences, University of Udine, via delle scienze 208, 33100 Udine, Italy

^3^ Istituto di Genomica Applicata, via Jacopo Linussio 51, Udine, 33100, Italy

* Corresponding authors:

Urska Vrhovsek Tel.: +39 046615140; fax: +39 0461615200 Email address: urska.vrhovsek@fmach.it

Gabriele Di Gaspero Tel.: +39 0432 629786; Email address: [digaspero@appliedgenomics.org](mailto:digaspero@appliedgenomics.org)

**Supplementary Information**

**Supplementary Figures**

**Figure S1** Differences in terpenoids between leaves undergoing *Rpv12*–mediated defence and controls during the course of infection. log_2_ ratio of the concentration of each compound in inoculated leaves versus controls.


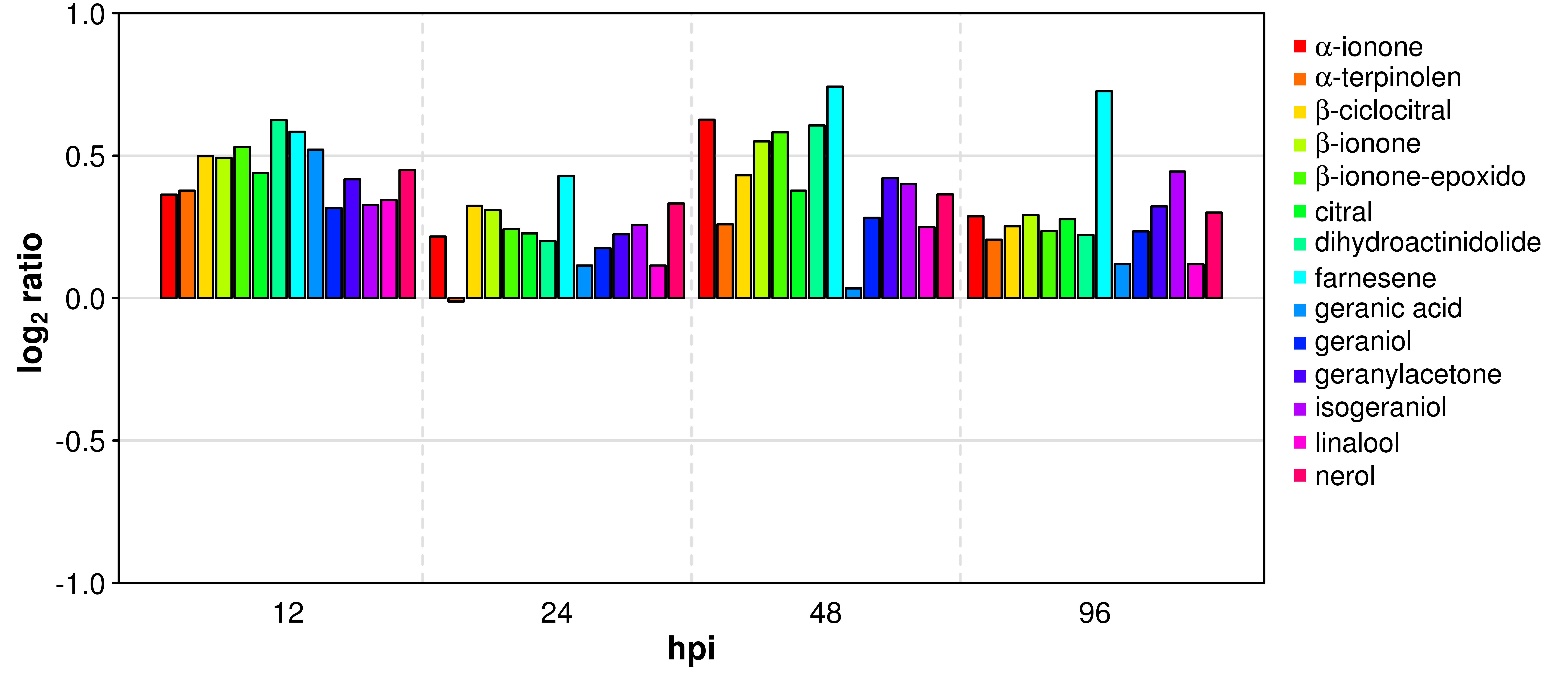


**Figure S2** Network of contrasting metabolite concentrations with highest statistical significance (t–statistic |t| >3) between leaves undergoing *Rpv12*–mediated defence and controls. **(a)** Distribution of t values for 175 metabolites at 4 time points. Metabolite concentrations with |t|>3 in **(a)** were used for drawing the network in **(b)**. The colour of the dot indicates the class of the compound: ● primary metabolites; ● lipids; ● phenols; ● volatile compounds. The colour of the connector indicates the significant time point of the relationship: **–** 12 hpi; **–** 24 hpi; **–** 48 hpi; **–** 96 hpi. The metabolite network in panel **(b)** was generated with the R package ggraph (Pedersen 2017, ggraph: An implementation of grammar of graphics for graphs and networks version 2.0.0 from CRAN. URL https://rdrr.io/cran/ggraph/).


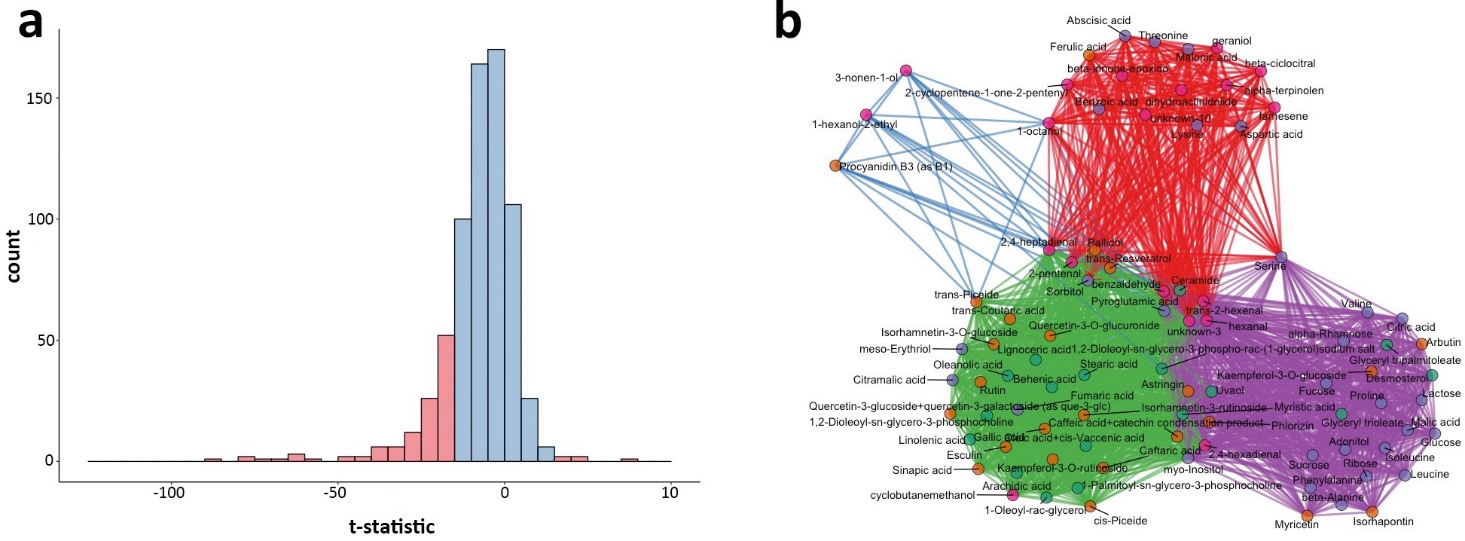


**Figure S3** Comparison of log_2_ fold changes in gene expression between leaves undergoing *Rpv12*–mediated defence (inoculated) and controls using (x–axis) normalized RNA levels against the expression of a housekeeping gene from qPCR data and (y–axis) FPKM estimates from RNA–Seq data.


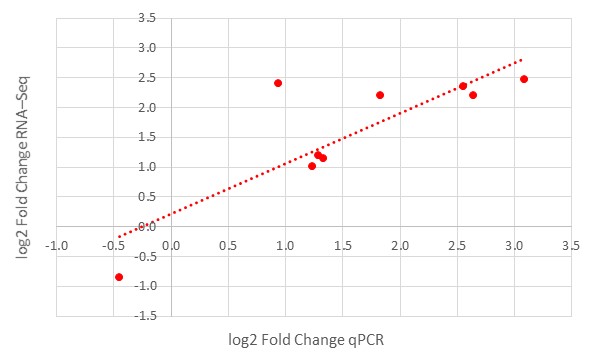


**Figure S4** Venn diagram of differentially expressed genes between leaves undergoing *Rpv12*–mediated defence (inoculated) and controls at only one specific stage of incubation or in common between two or more stages.


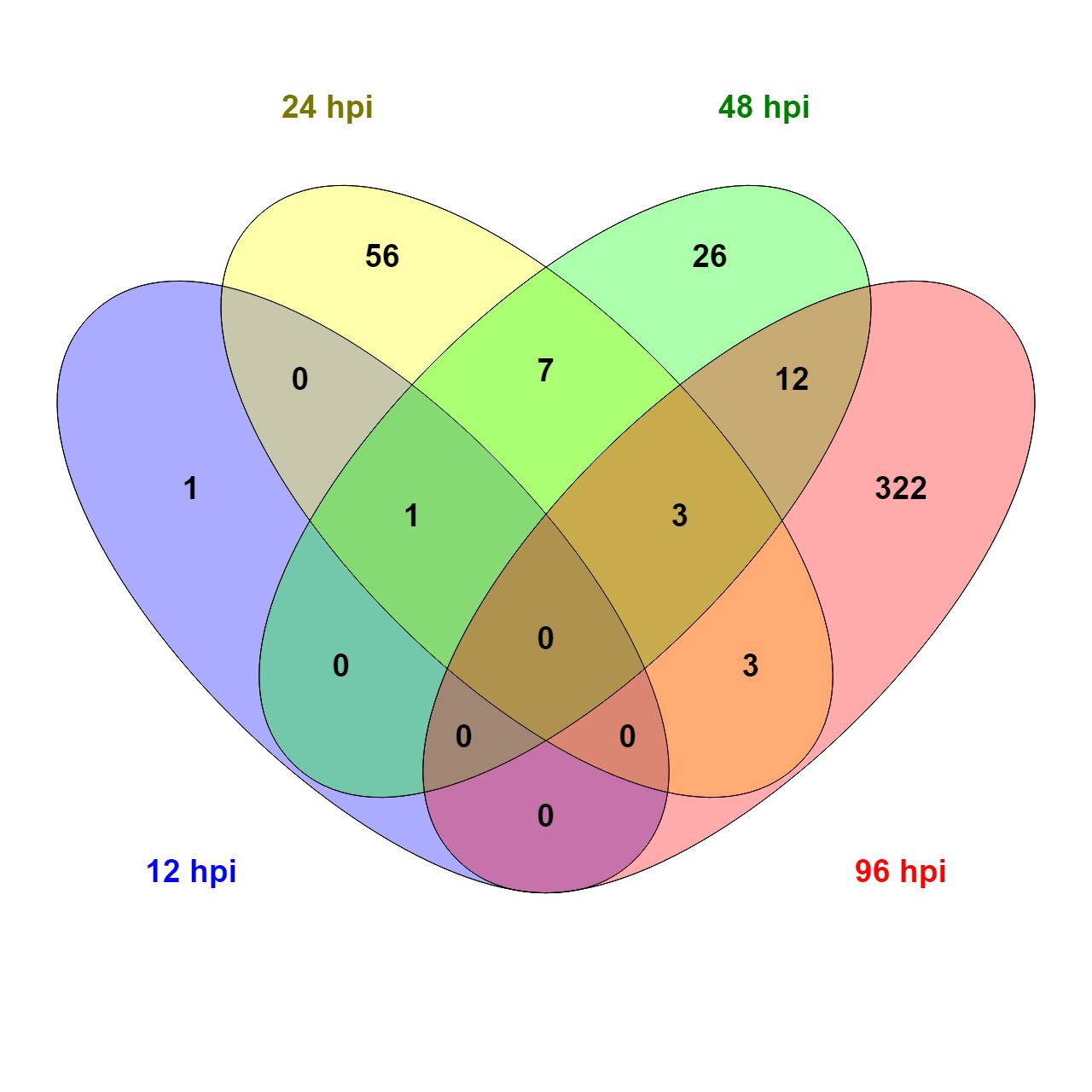

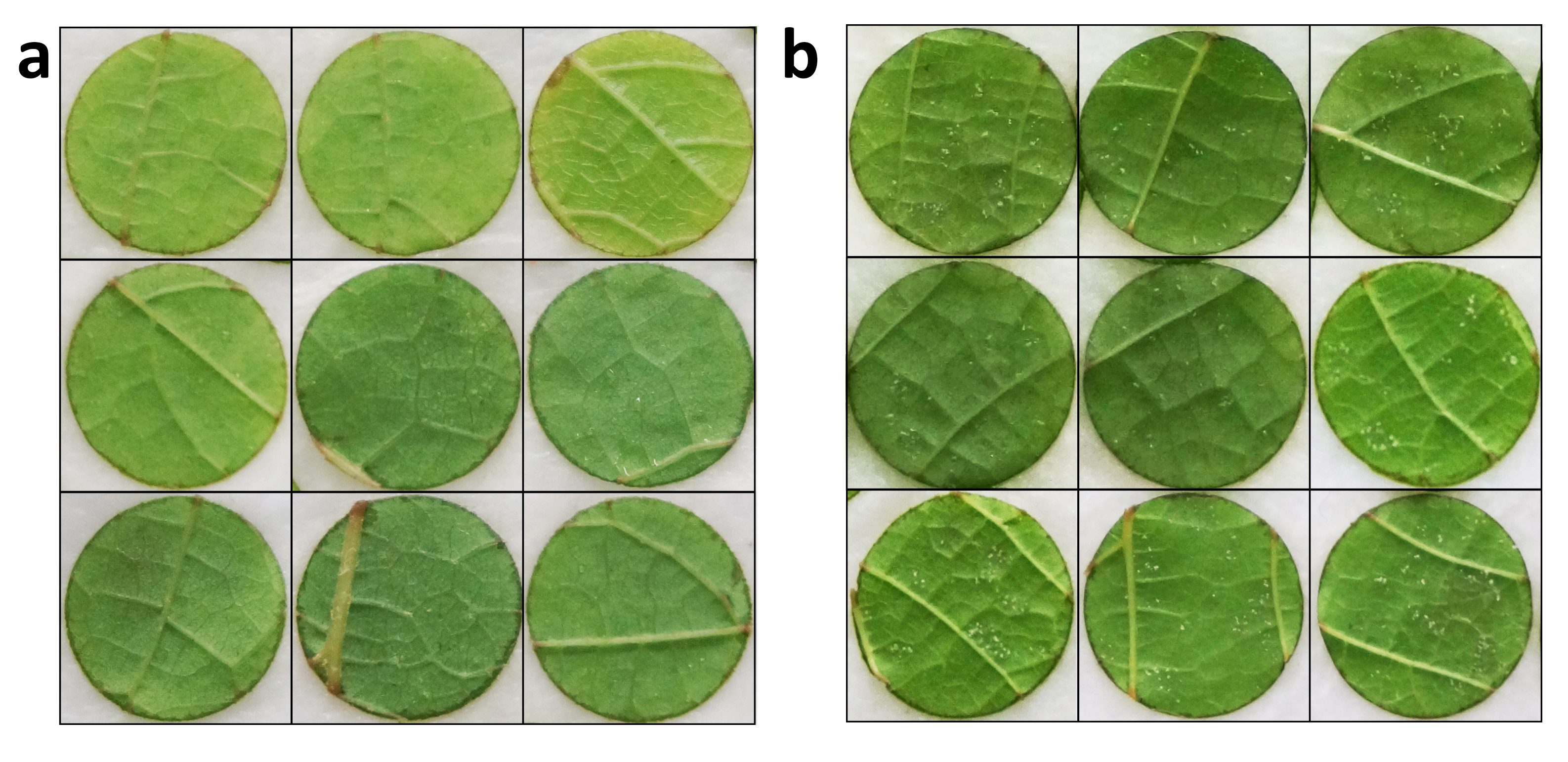


**Figure S5** Leaf discs photographed at 96 hpi using a Sony NEX-5R camera with default instrument settings and acquisition conditions. Images of the individual leaf discs were combined into a single image by juxtaposing. Borders of each individual photograph are demarcated by black margins. Processing changes of brightness and contrast were applied equally on (**a**) and (**b**) using the background, which was represented by moist paper in the Petri dishes, as an indicator for uniformity. Inoculated *Rpv12*–resistant leaf discs (**a**). Inoculated leaf discs of the sensitive variety ‘Pinot Noir’ (**b**).


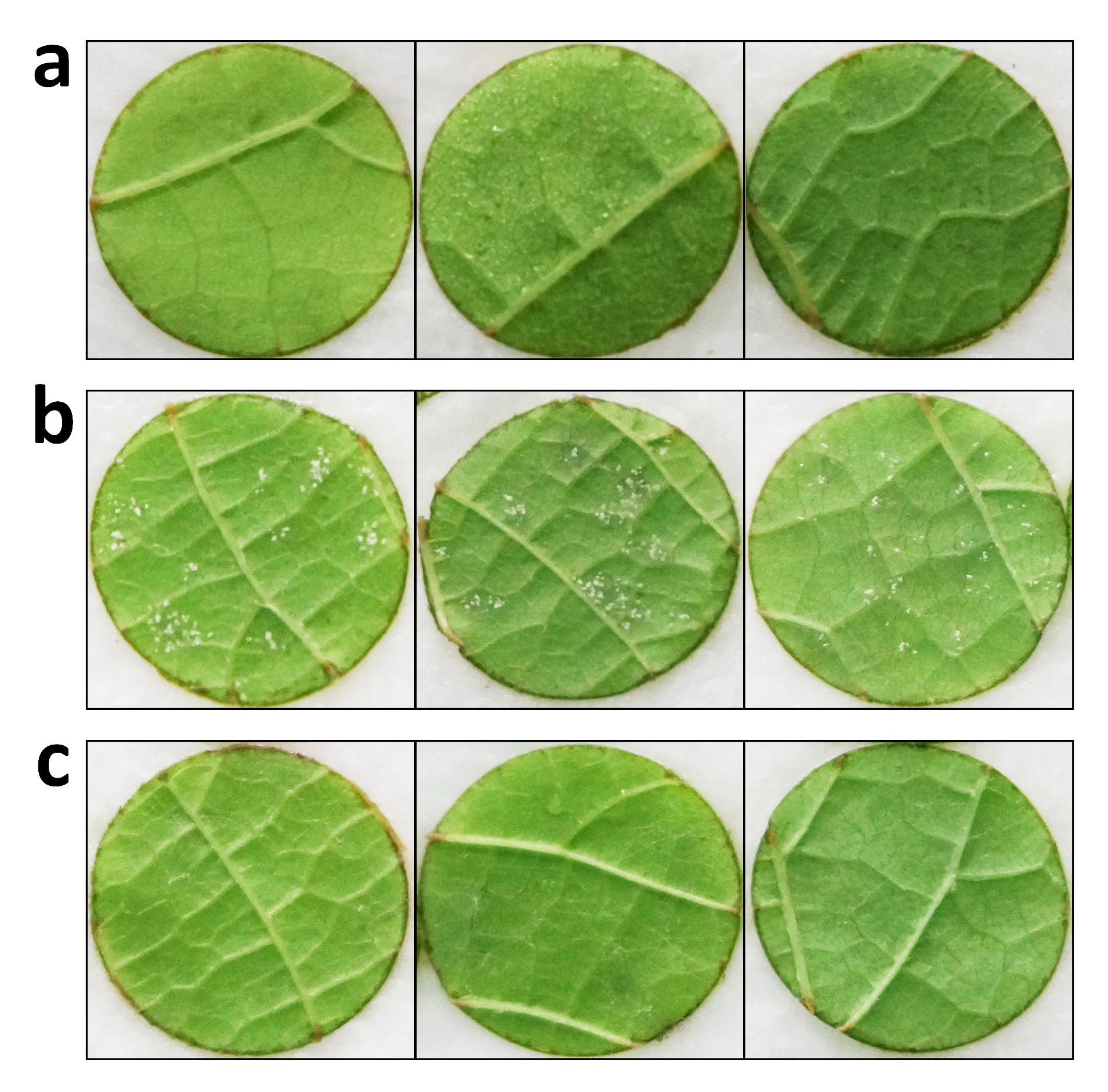


**Figure S6** Leaf discs photographed at 120 hpi using a Sony NEX-5R camera with default instrument settings and acquisition conditions. One representative leaf disc has been photographed from each biological replicate. Images of the individual leaf discs were combined into a single image by juxtaposing. Borders of each individual photograph are demarcated by black margins. Processing changes of brightness and contrast were applied equally on (**a**), (**b**) and (**c**) using the background, which was represented by moist paper in the Petri dishes, as an indicator for uniformity. Inoculated *Rpv12*–resistant leaf discs (**a**). Inoculated leaf discs of the sensitive variety ‘Pinot Noir’ (**b**). Mock–inoculated leaf discs of the sensitive variety ‘Pinot Noir’ (**c**).


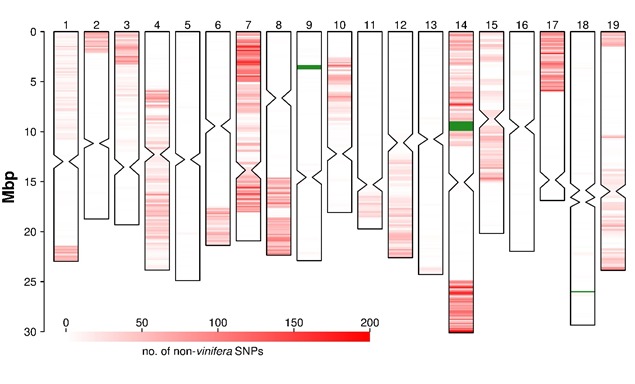


**Figure S7** Introgression map. Diagrams represent chromosomes. The y–axis indicates chromosome length in million base pairs (Mbp). The heat map indicates density of private SNPs detected in expressed genes of the genotype under study not shared with a comprehensive diversity–panel of the species *V. vinifera*. The color scale represents the number of non–*vinifera* SNP in non–overlapping windows of 100 Kb. Pure white segments indicate segments free of any non–*vinifera* introgression. Non–white segments indicate introgression from other *Vitis* species. Constrictions indicate the position of centromeric repeats. The green boxes and lines represent the physical intervals corresponding the genetic locus of *Rpv12* on the upper arm of chromosome 14, of *Rpv10* on the upper arm of chromosome 9 and of *Rpv3* on the lower arm of chromosome 18. The figure has been generated using R version 3.3. R Core Team (2017). R: A language and environment for statistical computing. R Foundation for Statistical Computing, Vienna, Austria. URL https://www.R-project.org/.


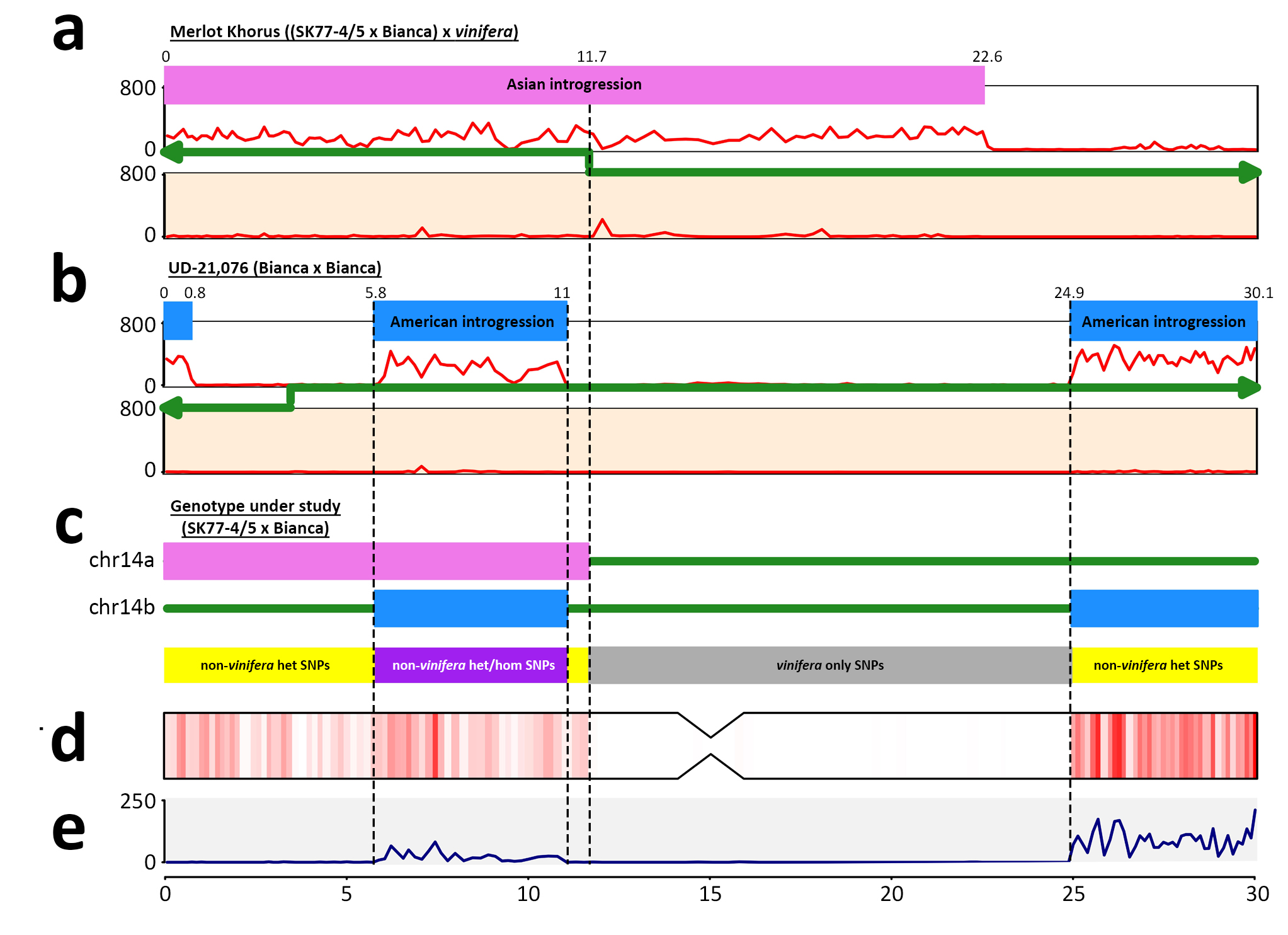


**Figure S8** Diagrams illustrating the genetic configuration of chromosome 14 in the genotype under study. Density plots of heterozygous (white background) and homozygous (shaded background) non–*vinifera* SNPs in ‘Merlot Khorus’ (**a**), a heterozygous variety at the *Rpv12* locus carrying the *Rpv12*–resistance haplotype donated by SK77–4/5 and a *vinifera* haplotype donated by the pure *vinifera* parent ‘Merlot Noir’, and in the breeding line UD–21,076 (**b**), which originated from selfing of ‘Bianca’. SNP density is expressed as the number of SNPs in non–overlapping 100–Kb windows of non–repetitive DNA in the *vinifera* reference genome sequence. Double headed green arrows in panels **a** and **b** show recombination in the ancestral homologues that have occurred before their transmission to the genotype under study. The recombination pattern in the homologs inherited from the SK77–4/5 lineage, carrying Asian *Vitis amurensis* introgression (highlighted by pink segment) and including the Rpv12–resistance haplotype at chr14:9.0–9.9 Mb, and from the ‘Bianca’ lineage, carrying American *Vitis* sp. introgression (highlighted by blue segments), were inferred from the density plots of non–*vinifera* SNPs in the genotype under study (**d**), their heterozygous (het) and/or homozygous (hom) state illustrated by yellow and violet segments in (**c**), and the absolute number of non–*vinifera* SNPs not shared with ‘Merlot Khorus’ in non–overlapping 100–Kb windows (**e**). The recombination breakpoint in (**b**) was drawn arbitrarily in the middle of the *vinifera* segment between two introgressions, but it may have occurred at any chromosomal coordinate between 0.8 and 5.8 Mbp. The color scale of the non–*vinifera* SNP density plot in panel (**c**) is the same as reported in Supplementary Fig. 7. The ruler at the bottom indicate Mbp. The figure has been generated using R version 3.3. R Core Team (2017). R: A language and environment for statistical computing. R Foundation for Statistical Computing, Vienna, Austria. URL https://www.R-project.org/.
